# Is Atopic Dermatitis Only a Skin Disease?

**DOI:** 10.3390/ijms24010837

**Published:** 2023-01-03

**Authors:** Alicja Mesjasz, Marta Zawadzka, Maciej Chałubiński, Magdalena Trzeciak

**Affiliations:** 1 Dermatological Students Scientific Association, Department of Dermatology, Venereology and Allergology, Faculty of Medicine, Medical University of Gdansk, 80-214 Gdansk, Poland; 2 Department of Developmental Neurology, Faculty of Medicine, Medical University of Gdansk, 80-952 Gdansk, Poland; 3 Department of Immunology and Allergy, Faculty of Medicine, Medical University of Lodz, 92-213 Lodz, Poland; 4 Department of Dermatology, Venereology and Allergology, Faculty of Medicine, Medical University of Gdansk, 80-214 Gdansk, Poland

**Keywords:** atopic dermatitis, comorbidities, epilepsy

## Abstract

Atopic dermatitis (AD) is a chronic, pruritic, inflammatory dermatosis that imposes significant patient and population burdens. In addition to the cutaneous signs and symptoms, growing evidence suggests that AD is systemic in nature. Certain diseases can possibly co-occur with AD as a result of coincidental exposure to similar environmental factors. However, it is also suspected that they are linked to the pathogenesis of AD through more complex genetic and immunological mechanisms, but these correlations remain less understood. It is of great need to seek explanations for the higher frequency of the number of cardiovascular, autoimmune, neurological, psychiatric, and metabolic disorders that have been observed in epidemiologic investigations among AD patients. Moreover, analysing the immunology of chronic inflammation and its correction, activation, or suppression may prevent the development of a variety of comorbidities. As comorbid diseases in patients diagnosed with AD may potentially go undetected, physicians should be aware of them.

## 1. Introduction

Atopic dermatitis (AD) is a common, chronic, inflammatory dermatosis characterised by persistent pruritus [1]. The clinical presentation consists of eczema-like eruptions, including erythema, papules, and exudative lesions of a specific location, depending on the patient’s age and varying extents of skin dryness [1]. Chronic or recurrent inflammation and scratching result in thickening and lichenification of the skin [1]. AD affects approximately 20% of children and 2–7% of adults worldwide, although the local prevalence varies by region [2].

Skin microbiota dysbiosis, altered immune response, environmental and genetic factors, and epidermal barrier disruption are five main factors engaged in the pathogenesis of AD. [1] Skewed immunity in the Th2-produced cytokines such as Il-4 and Il-13 lies on the basis of pathogenesis of AD across all endotypes [1]. In the acute phase of the disease, the T-helper (Th)1; Th22; and, to a lesser extent, Th17 cytokine-specific immune responses are enhanced [1]. In contrast, during the chronic phase, the response from Th2 and Th22 becomes more pronounced while that of Th1 decreases [1]. Additionally, various molecular patterns can be distinguished on the basis of factors such as ethnicity or age [3]. In terms of immunological distinctions between Asian AD and European/American AD, the first is characterised by greater activation of the Th17 axis and a less marked Th1 response [3]. Th1/Th17 attenuation and Th2/Th22 skewing characterise African/American AD, which is similar to paediatric AD, in which the Th2 response is more manifested and associated with atopic march, while the Th1 response is less pronounced [3]. To sum up, AD is strongly characterised by a Th2-driven immune response with additional activation of TH1, TH17, and TH22 cytokine pathways, depending on the disease phase and lasting, patients age and ethnicity [1]. It additionally opens the discussion on AD phenoendotypes and search for biomarkers [4]. This may not only play an important role in selecting the appropriate treatment but also in the possible increased co-occurrence of certain diseases in particular AD populations [4]. Chronic skin inflammation in AD patients results in skin microbiota dysbiosis and a rise in strains of pathogens such as *Staphylococcus aureus* that may account for more than 90% of skin microbiome in AD [5]. Exposure to environmental factors such as airborne or food allergens, pollution, infections, cosmetics, and strong detergents, as well as duration of breastfeeding, may contribute to a higher prevalence of AD [1]. Several loci have been associated with AD risk, with null mutations of the filaggrin (FLG) gene contributing highly to epidermal barrier deficiency being the strongest known genetic risk factors [6,7].

Although the presence of allergic comorbidities such as allergic asthma, rhinitis, and conjunctivitis is well established, less is known regarding the co-occurrence of AD and non-allergic comorbidities [8]. It is plausible, however, that the correlation among AD and comorbidities may be bidirectional and multifactorial [9]. Some co-morbidities may be related to the stress associated with living with chronic AD, while others may have their own unique pathomechanisms that are caused by or shared with AD [9]. Furthermore, some of these illnesses afflict children, whilst others occur in adults with long-standing illness, providing more evidence of the systemic nature of AD [8].

**Figure 1:**
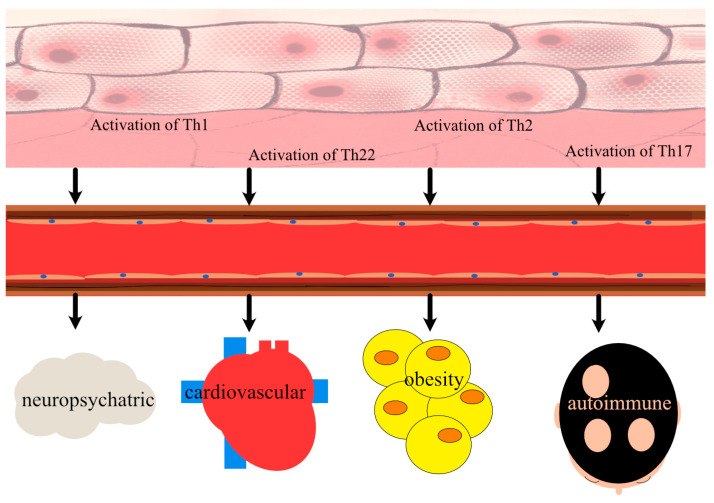
Comorbidities frequently associated with atopic dermatitis (AD). Even though AD is a skin disease, more is known about the co-occurrence of AD-related comorbidities that do not necessarily affect only the skin. The relationship between AD and comorbidities is likely to be bidirectional and complex. This list, however, is not exhaustive, and increasingly more diseases with a higher incidence among AD patients are being identified.

Our objective is to provide a summary of plausible explanations for why AD may be related with greater prevalence of certain cardiovascular diseases, neuropsychatric diseases (epilepsy, autism, attention deficit hyperreactivity disorder (ADHD), depression), autoimmune diseases (alopecia areata, vitiligo, rheumatoid diseases, type I diabetes (T1D)), and obesity (Figure 1). In addition, we wish to demonstrate the diversity and multiplicity of diseases that may be more prevalent in AD patients (or inversely) on the basis of epidemiological studies as well as underline the need for additional research into this phenomenon.

## 2. Materials and Methods

A comprehensive search of the literature using the PubMed (https://pubmed.ncbi.nlm.nih.gov/, accessed on 6 September 2022) electronic database using the search queries “(atopic dermatitis and comorbidities)”, “(atopic dermatitis and cardiovascular comorbidities)”, “(atopic dermatitis and neurological comorbidities)”, “(atopic dermatitis and psychiatric comorbidities)”, and ‘’(atopic dermatitis and autoimmune comorbidities)” was performed in the second week of September 2022 from the database inception to the 16 September 2022. Further research were used to conduct a comprehensive search of literature: “(atopic dermatitis and vascular inflammation)”, “(atopic dermatitis and coronary artery disease)”, “(atopic dermatitis and artherosclerosis)”, “(atopic dermatitis and thrombosis)”, “(atopic dermatitis and neuro-immune crosstalk)”, “(atopic dermatitis and epilepsy), “(atopic dermatitis and autism)”, “(atopic dermatitis and attention deficit hyperactivity disorder), “(atopic dermatitis and depression)”, “(atopic dermatitis and alopecia areata), “(atopic dermatitis and vitiligo)”, “(atopic dermatitis and chronic urticaria)”, and “(atopic dermatitis and celiac diseases) were performed in the third week of September 2022 from the database inception to the 1 October, and “(atopic dermatitis and type I diabetes)” and “(atopic dermatitis and obesity)” were performed in the third week of September 2022 from the database inception to the 28 September 2022. After the initial search, the inclusion and exclusion criteria were applied to the titles and abstracts. On the basis of the analysis of titles and abstracts, we included articles on the causes of the increased co-occurrence of atopic dermatitis (genetic factors, immunological factors, exposure to modifiable risk factors).

We excluded non-related records, non-English manuscripts, personal opinions, and duplicates at this stage. The remaining part were deemed suitable for full-text viewing. After reading the complete manuscripts, some were omitted (not relevant, not original, and not providing information concerning earlier mentioned topics).

## 3. Discussion

### 3.1. AD Patients Behaviours That May Enhance Comorbidity Prevalence

It is well established that regular physical activity aids in the primary and secondary prevention of a number of chronic diseases including obesity, depression, and cardiovascular diseases [10]. Adult AD was associated with decreased moderate to vigorous and overall physical activity in the USA, according to the Silverberg et al. study [11,12]. There are several potential causes for this: patients with eczematous skin lesions on their palms and soles might find it difficult to participate in a variety of activities, but also elevated skin temperature and perspiration are known flare triggers [11,13]. Furthermore, sleep disturbance and depression, which may occur in AD patients, may make it more difficult to maintain a regular exercise routine [11,12,13]. Sleeplessness and social shame caused by disfiguring skin lesions may contribute to the development of conditions such as depression and anxiety among AD patients [14,15,16]. Positively associated with AD and food allergy but also obesity was prenatal and early life antibiotic exposure [17,18,19,20]. AD is also linked to an increased incidence of eating disorders, with bulimia nervosa and binge eating disorder being the most prevalent [21]. It is well established that binge eating disorder is associated with higher incidence of obesity [22]. Incorrect administration of a systemic treatment for AD may also potentially be a factor. Glucocorticosteroids, for instance, can cause side effects such as weight gain, sleep disturbance, mood changes, and hyperglycemia, whereas cyclosporine A can cause renal impairment and high blood pressure [23]. Furthermore, AD patients communicate with physicians more frequently, which may facilitate the detection of diseases that might otherwise go undetected.

### 3.2. Cardiovascular Diseases

Individual studies have reached different conclusions regarding the link between AD and cardiovascular diseases [24,25,26]. For instance, in the U.S. study, 1-year history of AD was associated with a higher prevalence of coronary artery disease (CAD), angina pectoris, myocardial infarction (MI), stroke, and peripheral vascular disease (PVD) [24]. In the German study, AD patients had an elevated risk of angina pectoris, hypertension, and PVD, but not of MI or stroke [25].

**Figure 2:**
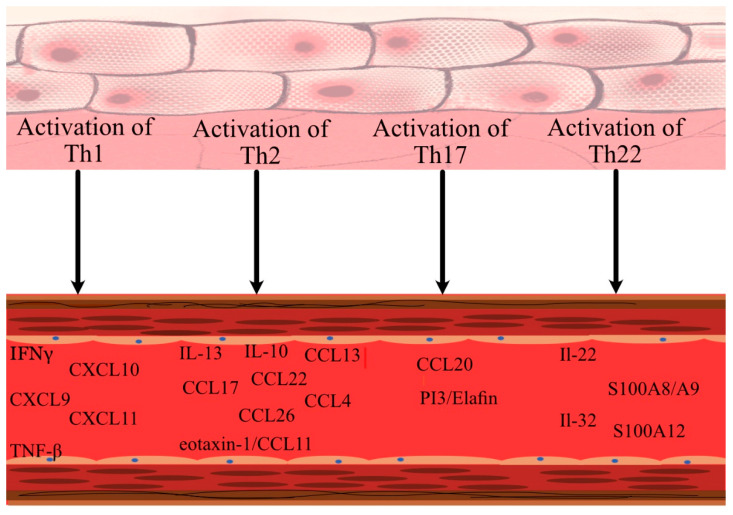
Upon activation of critical T helper (Th) cells involved in the pathogenesis of AD, a number of cardiovascular biomarkers are upregulated. INF-γ—interferon gamma, TNF-β—tumour necrosis factor beta, CXCL—C-X-C motif ligand, Il—interleukin, CCL—C-C motif chemokine ligand, PPi3/elafin—peptidase inhibitor 3/elafin, S100A12—calgranulin C, S100A8/A9 (=MRP8/14)—myeloid-related protein 8/14.

Variation in endotypes and lifestyle choices between nations may account for the observed differences [26,27]. While the higher cardiovascular risk in psoriatic patients as, at least to some extent, a result of elevated levels of immune and cardiovascular proteins is a well-established concern, the data on AD are less researched and still emerging [28]. Firstly, AD patients and psoriatic patients share an increase in several inflammatory and cardiovascular risk markers in their blood serum, including T helper (Th)1 (interferon gamma (IFN-γ), tumour necrosis factor alpha (TNF-α), and Th17 (C-C motif chemokine ligand 20 (CCL20)) (Figure 2) [28]. Others, however, proved to be uniquely significantly elevated in the blood of AD patients; these included some atherosclerosis (fractalkine/CX3C motif ligand 1 (CX3CL1), macrophage-colony-stimulating factor (M-CSF)), T-cell development and activation (cluster of differentiation 40 ligand (CD40L), interleukin (IL)-7, CCL25), and angiogenesis (vascular endothelial growth factor A (VEGF-A)) markers [28]. Atypically, AD patients demonstrated a positive connection between Th2-related cytokines (CCL17, CCL22), Th22-related cytokines ((IL-22, myeloid-related protein (MRP8/14), and IL-32), and vascular inflammation, especially given that atherosclerosis is a Th1-dominated illness and the role of Th2-produced cytokines remains contentious (Figure 2) [29]. Serum indicators positively correlated with severity of AD as assessed by the scoring atopic dermatitis (SCORAD) scale, but were unrelated to body mass index (BMI) [28,29]. Additionally, dupilumab treatment reduced the levels of cardiovascular indicators in the skin and blood of AD patients and significantly modified the atherosclerosis genes [29,30].

There also appears to be a positive correlation between the values of cardiovascular indicators and age, as patients over the age of 60 showed greater levels of several markers of arthrosclerosis, cardiovascular risk, cell adhesion, and apoptosis compared to younger patients with AD and age-matched controls without AD [31]. Nevertheless, multiple cardiovascular markers were also found to be raised in pediatric AD and they included E-selectin, an endothelial cell adhesion molecule, and several matrix metalloproteinases (MMP), which are proteins engaged in tissue remodelling [32]. Observations show that immunoglobulin E (IgE) may also play a role in cardiovascular homeostasis and disease; nevertheless, this idea is relatively new and poorly understood [33].

Some research indicates that AD increases blood platelet activation and oxidative stress while decreasing fibrinolysis, which may contribute to the development of thrombosis; however, in other research, the function of platelet aggregation was not impaired in AD patients [34]. In contrast, increased activity of proinflammatory mast cells and tryptases reduced the risk of thrombosis by tryptase-mediated degradation of fibrinogen, a thrombosis mediator, and creation of a complex between heparin and tryptase, resulting in anticoagulation [34,35]. The therapeutic significance of mentioned correlations is unknown, and there is currently no clear evidence that patients with AD require more extensive cardiovascular monitoring or therapy than is suggested for the general population [36]. Nevertheless, there are some attempts to implement screening for cardiovascular comorbidities [37].

### 3.3. Neurologic and Psychiatric Diseases in AD

#### 3.3.1. Neuro-Immune Crosstalk in AD

The genesis and maintenance of atopic itch are dependent on crosstalk between the neurological system, cutaneous immune response, and keratinocytes [38]. Degranulation of mast cells triggered by allergens and allergen-specific IgE as well as cytokines results in the release of histamine, which can bind with C- and A-delta nerve fibres via histamine 1 (H1) and H4 receptors [38,39]. However, this mechanism plays a minor role in chronic pruritus, in contrast to histamine-independent pathways [38,39]. Particular members of the polymodal transient receptor potential (TRP) ion channel superfamily, which plays a crucial role in inflammation, itch, and pain, are overexpressed and show heightened sensitivity in AD skin [40]. They include TRP such as transient receptor potential V member 1 (TRPV1), transient receptor potential ankyrin 1 (TRPA1), TRPV3, and TRPV4 [40]. Through direct activation interleukin-31 receptor A (IL-31RA) on TRPV1+/TRPA+ sensory neurons in the skin, Th-2 derived IL-31 promotes itch [41].

Other AD-associated cytokines such as Il-13 and thymic stromal lymphopoietin (TSLP) can act similarly with protease-activated receptor-2 (PAR2)-regulated TSLP secretion of keratinocytes regulated by calcium release-activated calcium modulator 1/nuclear factor of activated T-cells (ORAI1/NFAT) calcium signalling pathway, being an example showing that keratinocytes can also directly communicate with sensory neurons [42]. Moreover, in vivo, increased levels of TSLP work directly on CD4+ T cells to cause the establishment of IL-13-single-positive (IL-13-SP) and IL-4+IL-13+ double-positive populations in lymph nodes modifying the function of immune system [43]. Harmful stimuli and stress via, for instance, upraised levels of nerve growth factor (NGF), can uprise the amount of nerve fibres that secrete neuropeptides such as neuropeptide Y (NPY), substance P, calcitonin-gene regulated peptide (CGRP), or brain natriuretic peptide (BNP) [44]. These factors regulate the function of various inflammatory cells including mast cells and release of cytokines [44].

#### 3.3.2. Epilepsy

According to a study conducted on 35,312 patients with AD and their age- and gender-matched controls in Taiwan, AD was found to be associated with a greater risk of epilepsy development [45]. Similar results were observed in a study conducted on the United States (US) population [46].

Neuroinflammation is one of the processes involved in the pathogenesis of epilepsy [47]. In turn, microglia and astrocytes are the main factors involved in neuroinflammation [39]. Microglial activation is a response to pro-inflammatory mediators released from immune cells, including mast cells [39]. Mast cells act as skin-resident immune cells, which are potential sources of inflammatory mediators in AD [39]. The mast cells are also present in various part of the brain: in the postrema area, choroid plexus, and parenchyma of the hypothalamic thalamus [39]. In vitro, it has been found that mast-cell-derived tryptase can activate proteinase-2-activated receptors (PAR-2) in microglia, which triggers the release of pro-inflammatory mediators such as TNF-α, IL-6, and reactive oxygen species (ROS). [39] Consequently, IL-6 can induce the release of IL-13 from mast cells and affect Toll-like receptor (TLR)2/TLR4 expression [39]. In the process of neuroinflammation, excessive levels of inflammatory mediators may affect neurogenesis, neurodegeneration, and permeability of the blood–brain barrier (BBB) and possibly disrupt the balance of neurotransmission, causing many neurological disorders, including epileptic seizures [39].

In addition to the influence of the inflammatory reactions associated with AD on the nervous system, the role of the peripheral and central nervous systems in the development of AD symptoms seems to be extremely important [48]. Recent research shows that neurotransmitters and neuropeptides released from peripheral neurons cause mast cell degranulation, and, in turn, bioactive compounds released from mast cells stimulate the function of nerve fibres [48]. This neural loop in peripheral tissues is involved in causing allergic inflammation [48].

Silverberg et al. found in mice study that a single tonic–clonic seizure induces IL-4- and IgE-negative T and B cells to migrate to the brain and transform into Il-4+ and IgE+ cells, respectively [49]. There is an increase in Il-4 in the serum of patients after seizures, indicating that this cytokine may be a natural response of the body to injury [50,51]. Nevertheless, it is worth mentioning that, due to the multimodal action of cytokines, IL-4 can play anti-inflammatory or pro-inflammatory roles in different environments [50,51]. IL-4 therefore likely plays a significant role in epileptogenesis and the physiopathology of epilepsy [50,51]. IL-33 on the other hand seems to have a rather protective role, whereas the role of IL-13 is debatable [50,52]. Genetics can also be involved in the positive correlation between AD and neuropsychological comorbidities, with the deletion of chromosome 22q13.2 manifesting as AD, epilepsy, mental retardation, and autism spectrum disorder (ASD) [45,53].

#### 3.3.3. Autism

The outcomes of a review of 18 studies reveal a link between ASD and AD, indicating that individuals with ASD have a higher risk of presenting with AD when compared to controls, and inversely [54]. Moreover, atopic disorders, notably comorbid AD, are correlated with a rise in the severity of ASD symptoms [55].

Autism spectrum disorder (ASD) is another comorbid condition that may occur alongside AD. ASD has an unknown aetiology, yet the exposure to the combination of genetic and environmental factors, both prenatally and postnatally, is likely to contribute to the pathogenesis of ASD by influencing brain development [56]. One of the environmental factors considered in the pathogenesis of ASD is neuroinflammation [56]. Abnormally elevated levels of IL-1β, IL-6, IL-8, and IL-17 have been found in studies of in patients with ASD of all ages [56]. In mouse models, ASD behaviour has been observed in the offspring of mice exposed to lipopolysaccharide, Il-17, and polyinosine polycytidylic acid during pregnancy [56]. Researchers also highlight the role of neural cellular adhesion molecule (NCAM)1, which appears to regulate nuclear factor kappa B (NF-κB) transcription in neurons, altering pro-inflammatory signalling [56]. Moreover, NCAM1 and contactin-1 can mediate the proliferation of astrocyte and oligodendrocyte precursors, altering the neuroimmune response [56].

A meta-analysis that included 12 studies displayed a positive correlation between food hypersensitivity and ASD, with subjects under the age of 12 being more susceptible to this association [57]. Mice with an experimentally induced cow’s milk allergy exhibited autistic-like behavioural abnormalities, an increase in several biomarkers, and enhanced mTOR brain signalling in ASD [58]. Nonetheless, additional long-term studies on large cohort groups are required, and scepticism seems appropriate. Furthermore, because both epidermis and neural tissues originate from the embryonic neuroectoderm, it is possible that the presence of neuro- and epidermal toxicity at the onset of AD and ASD reflects the shared susceptibility of the brain and epidermis in the pathogenesis of both AD and ASD as studied on a mice model [59].

#### 3.3.4. Attention Deficit Hyperactivity Disorder

Attention deficit hyperactivity disorder (ADHD) has a higher prevalence in children diagnosed with AD in the studies performed in the USA, Germany, Iran, and Korea [51,60,61,62]. The more atopic diseases a patient has, the more severe their ADHD, according to two meta-analyses involving 25,000 individuals [63]. Moreover, there appears to be a higher risk of atopic disease development among siblings of children with ADHD, suggesting a common genetic factor [64].

Possible contributors to the development of ADHD in later childhood include the suspected role of sleeping problems in infancy but also early exposure to sedative H1-antihistamines that can cross the BBB [61,65]. The hypersecretion of inflammatory mediators including the Th1-, Th2-, and Th17-derived cytokines that may alter the maturation process and neuroactivity of the prefrontal cortex (PFC), and ACC is one of the most accepted explanations for the coexistence of these two disorders [62,63,66].

#### 3.3.5. Depression

An increased risk of depression, suicidality, parental depression, and antidepressant use were all concluded to be related with having AD in a meta-analysis combining 36 studies [67,68]. It is important to note, however, that individual studies frequently deliver contradictory results, and this held true for all diseases discussed in this review. Similarly to adults, a positive correlation between AD and depression in children was discovered; however, the estimate was lower [68]. Given that one in six patients with AD has depression and one in four experience depressive symptoms, the problem appears to be of significant concern [67]. Furthermore, dupilumab improves patients’ self-reported symptoms of depression and anxiety, supporting a severity-dependent connection existing between AD and depression [67,68,69].

The most widely recognised mechanism that could explain link between AD and psychiatric disorders is the overactivity of the hypothalamic–pituitary–adrenal (HPA) axis and the sympathetic nervous system (SNS) [70,71]. Increased corticotropin-releasing hormone (CRH) and the associated glucocorticoid receptor resistance contribute to the upregulation of inflammatory cytokines and the maintenance of inflammation, whereas the activation of brain microglia by the same cytokines is a proposed mechanism underlying concomitant depression [70]. Certain subtypes of depression were discovered to be significantly linked with hypercortisolemia [72]. IgE-mediated degranulation of mast cells may elicit hypothalamic–pituitary–adrenal responses via a centrally released histamine, possibly resulting in an increase in CRH and, consequently, an increase in cortisol levels reported in some studies on AD patients [70,73,74].

The prevalence of mental retardation, psychological development disorders, memory impairment, and behavioural and emotional disorders among AD children were also reported [60,75].

### 3.4. Autoimmune Diseases

#### 3.4.1. Alopecia Areata

Patients with alopecia areata (AA), notably alopecia totalis or alopecia universalis, and vitiligo, particularly early onset, had a considerably higher risk for AD, according to a meta-analysis comparing 16 observational studies with vitiligo and 17 with AA [76].

The increased frequency of FLG mutations in AA patients with a history of AD implies a genetic connection [77]. Furthermore, recent research indicates that AA is engaged not only in the Th1-driven process, as it was believed, but also in the Th2 route [36]. Type 2 immunity may contribute to the development of AA in patients presenting extrinsic AD [36]. Studies indicate further connections between atopy and AA, such as increased IgE, regardless of an atopic background [78]. A clinical trial assessed the safety of dupilumab, an anti-IL-4Rα antibody that inhibits the release of Il-4 and Il-13, the key cytokines released by Th2 in AD, in AA after several cases of hair growth were reported in the literature following the administration of dupilumab to patients with AA and AD [78]. On the other hand, dupilumab has also been observed to induce new-onset AA [79]. Baricitinib is, however, the drug registered in both Europe and the United States for severe alopecia areata in adults and in Europe for moderate to severe AD in adults [80,81]. By inhibiting the downstream signals of interferon, IL-4, IL-13, baricitinib improves Th1-driven AA and AD, and types of AA in which Th2 cytokines respond also notably enhanced [82].

#### 3.4.2. Vitiligo

According to a meta-analysis, AD was associated with higher odds of vitiligo prevalence [83].

Vitiligo is an autoimmune epidermal condition characterised by the destruction of melanocytes and depigmentation of the skin, primarily due to their heightened vulnerability to oxidative stress and the subsequent activation of innate immunity before adaptative immunity [84]. The pro-inflammatory condition of AD, as proposed by Silverberg et al., may predispose toward melanocyte destruction, and scratching pruritic AD lesions may provoke a Koebner effect in vitiligo [85]. Genetic abnormalities in major histocompatibility complex (MHC) class I and III along with vitamin D receptor polymorphisms may be the predisposition to the development of both AD and vitiligo [6,85].

#### 3.4.3. Rheumatoid Diseases

The metanalysis comparing 13 studies concluded that patients with AD have a significantly higher risk of incidence of rheumatoid arthritis (RA) [86]. Similar findings were seen for several other rheumatoid diseases including ankylosing spondylitis (AS), systemic lupus erythematosus (SLE), and Sjögren’s syndrome [83].

Both AD, especially intrinsic or acute, and RA share common immunopathogenesis in which cytokines produced by Th1 including TNFα and Th17 cells both play important roles [1,3,8,9,10,11,12,13,14,15,16,17,18,19,20,21,22,23,24,25,26,27,28,29,30,31,32,33,34,35,36,37,38,39,40,41,42,43,44,45,46,47,48,49,50,51,52,53,54,55,56,57,58,59,60,61,62,63,64,65,66,67,68,69,70,71,72,73,74,75,76,77,78,79,80,81,82,83,84,85,86,87,88,89]. Furthermore, at baseline, patients with AD already have a higher level of autoreactivity as compared to the general population [88,89]. It could be for instance explained by impaired epithelium that sensitise the immune system to autoimmune diseases [88,89]. Several autoantibodies associated with SLE, such as antinuclear antibodies, anti–Sjögren’s-syndrome-related antigen A (anti-SSA), and anti-ribonucleoprotein (anti-RNP) were detected in the blood of AD patients [90]. However, the elevated levels of circulating IgE seen in certain populations of SLE patients were not related to an increased incidence of atopic illness in this patient cohort [91].

Epigenetic changes along with gene variations enhancing susceptibility to both rheumatoid diseases and AD are also a possibility, and therefore conflicting results have been found in identifying them [7,86,92,93]. Non-receptor protein tyrosine phosphatase (PTPN)22 and human leukocyte antigen (HLA)DRB1 are some of the examples [86,92,93]. Furthermore, a functional polymorphism in IL6R, for instance, has been demonstrated to raise the risk for AD and asthma while having a protective effect on RA [94]. It demonstrates that the opposite direction of genetic effects in each of these diseases also occur [94]. Similar medications are used in both RA and AD, such as baricitinib and upadacitinib, which are approved in Europe and the USA for both diseases [81,87,95].

#### 3.4.4. Type I Diabetes

The epidemiological studies on the co-prevalence of AD and type I diabetes (T1D) are inconclusive, indicating a predominantly negative association [96,97,98].

Th1 that produce interferon lambda (IFN-λ) and IL-12, which are associated with islet cell destruction, are critical to the pathophysiology of T1D, whereas Th2 is believed to have a protective role [97]. Th17 cells and regulatory T cells (Tregs) are also involved in the pathogenesis of both AD and T1D since Th17 cells promote atopy and autoimmunity while Tregs exert a suppressive effect [97]. It can be speculated that an increased risk of Th2 diseases following the onset of Th1 diseases is responsible for the higher co-occurrence of both diseases, overcoming any potential inverse connections during the preclinical phase of the Th1 disease [96]. The hygiene hypothesis is one of the postulated processes assumed to be responsible for the rising rates of both T1D and allergy illnesses, since affluence is associated with less exposure to infectious agents, which may increase the population’s vulnerability to both conditions [99]. One study found a protective association between the presence of diabetes-related autoantibodies and the subsequent development of asthma and AD [100]. Furthermore, after being diagnosed with one illness, children have increased contact with health services, which may raise their chances of being diagnosed with atopic conditions [101]. However, the burden of diabetes management may cause parents to minimise the significance of atopic conditions, resulting in their underreporting [101]. An inverse link between genetic variables for AD and T1D was also attempted to explain the negative correlation between the co-occurrence of these diseases [94,102].

### 3.5. Obesity

In North America and Asia, but not in Europe, a meta-analysis of 30 studies performed on all age groups revealed a connection between overweight along with obesity and AD [103]. However, this discrepancy may also be attributable to varying definitions of obesity and worldwide variations of AD prevalence and different endotypes across populations [104].

Firstly, adipose tissue is not simply a passive storage depot for lipids—it plays a significant role in various endocrine, metabolic, and inflammatory processes [105]. Uncontrolled adipocyte hypertrophy promotes the secretion of proinflammatory adipokines such as leptin or resistin and decreases the release of anti-inflammatory ones including adiponectin, resulting in immune cell recruitment [106]. The levels of adiponectin and resistin were inversely associated to the severity of eczema as measured by the SCORAD scale, according to a study evaluating the potential utility of adipokines as AD severity biomarkers [107]. In addition, the levels of adiponectin and resistin were lowered in adult patients with extrinsic AD, while leptin levels were significantly elevated [107]. Many cytokines, particularly Th1-derived cytokines such as TNF-α, IFN-γ, IL-6, and IL-2, are increased when obesity is present, resulting in chronic low-grade inflammation that may predispose individuals to hypersensitive reactions [103,108].

Obesity has been proven to have an impact on an immune profile of AD, altering the immune response in a mouse model with AD from a classical Th2-predominant disease to a more severe disease with additionally pronounced Th17 inflammation [109]. In addition, Th2-cytokine-targeting biologic treatments effectively protected lean mice from illness aggravation and Th17 skew but aggravated the disease in obese mice [109]. In these mice, T cells exhibited decreased peroxisome-proliferator-activated receptor (PPAR) activity, a transcription factor important for controlling transcriptional pathways in Th2 cells [109]. Thiazolidinediones (TZDs), an FDA-approved class of PPAR agonists used to manage type 2 diabetes, improved the outcomes of anti-IL-4/IL-13 for treating AD in obese mice by reducing immunological misfiring [109]. In general, obesity was proven to increase transepidermal water loss (TEWL), which is critical to AD pathogenesis [105]. Potential explanations include elevated blood pressure, decreased water permeability in the cutaneous barrier, and an increase in sweat gland reactivity in obese patients [105,110].

## 4. Conclusions

AD appears to be associated with multiple comorbid allergic, cardiovascular, mental health, neurologic, autoimmune, and metabolic conditions. It is essential to determine the extent to which this coexistence is linked to exposure to, often modifiable, risk factors, as well as genetics and immune dysregulations. Additionally, analysing the immunology of chronic inflammation whose correction, activation, or suppression would potentially aid in preventing the development of a variety of comorbidities is also key. Further research should be conducted on populations that are maximally vast and diversified to study predispositions depending on different phenoendotypes. Furthermore, it would be desirable to examine biomarkers that could be used to assess the likelihood of comorbidities in different populations. Nonetheless, clinicians should be aware of non-allergic comorbidities associated with AD and attempt to detect them as they can be potentially undiagnosed.

## Data Availability

All the data can be found in the PubMed database—https://pubmed.ncbi.nlm.nih.gov/ (accessed on 6 September 2022) or under the links cited of cited websites.
